# Electrocatalytic C─C Bond Formation Via Cyanide Interception of CO_2_ Reduction Intermediates

**DOI:** 10.1002/advs.77346

**Published:** 2026-08-21

**Authors:** Gongbo Liu, Liuru Fang, Dayu Zhu, Aimin Li, Haoming Yu, Chunlu Ding, Yuen‐Leong Chow, Mengjie Liu, Jianqi Geng, San H. Thang, Jie Zhang

**Affiliations:** ^1^ School of Chemistry Monash University Clayton Melbourne Australia; ^2^ School of Chemistry and Chemical Engineering Beijing Institute of Technology Beijing China; ^3^ ARC Research Hub For Carbon Utilisation and Recycling Monash University Clayton Australia; ^4^ ARC Centre of Excellence for Green Electrochemical Transformation of Carbon Dioxide Monash University Clayton Australia

**Keywords:** C‐C coupling, CO_2_ reduction, electrolysis, glycolonitrile

## Abstract

Electrochemical CO_2_ reduction offers a sustainable route to convert greenhouse gas into high‐value‐added chemicals, yet product distributions remain largely limited to simple C_1_‐C_3_ molecules. Here, we report a new reaction in which CO_2_ reduction intermediates undergo direct C‐C coupling with an external carbon nucleophile under electrochemical conditions. Using cobalt phthalocyanine supported on multi‐walled carbon nanotubes (CoPc/MWCNT) as a catalyst, cyanide ions intercept deeply reduced C_1_ intermediates to produce glycolonitrile at 4°C with a faradaic efficiency (FE) of up to 6.6%. Combined electrochemical analysis, control experiments, and density functional theory calculations identify *CH_2_O as the key coupling intermediate, revealing a C‐C coupling mechanism fundamentally different from conventional coupling pathways in CO_2_ electroreduction to synthesize C_2+_ compounds. Extending this concept to a three‐component reaction involving hydroxylamine (NH_2_OH) enables electrocatalytic synthesis of glycine under ambient pressure with a FE of 2.8%. This work establishes a new strategy for constructing complex carbon skeletons directly from CO_2_ and external nucleophiles, expanding the synthetic scope of electrochemical carbon conversion.

## 1 Introduction

Electrochemical carbon dioxide reduction reaction (eCO_2_RR) has emerged as a promising strategy to enable carbon‐neutral and establish a sustainable carbon cycle [1, 2]. So far, the majority of eCO_2_RR studies have focused on the production of C_1_‐C_3_ fuels and bulk chemicals, such as carbon monoxide [3, 4], formate [5, 6], methanol [7, 8], and ethanol [9, 10, 11, 12]. While these products are industrially relevant, their limited economic value and highly competitive markets impose intrinsic constraints on the broader impact of CO_2_ electroreduction [13].

In addition to these products, recent advances have revealed that highly reactive C‐centered intermediates generated during eCO_2_RR, such as *CHOH [14, 15], *CH_2_O [16], and *CCO [17, 18], can be intercepted by external nucleophiles to directly construct high value‐added organic molecules [19, 20]. This emerging strategy connects electrochemical CO_2_ conversion with synthetic chemistry, enabling the upgrading of CO_2_ into structurally complex molecules using renewable electricity. Notably, the incorporation of N‐containing nucleophiles has enabled the electrosynthesis of amides [16], amino acids [19], urea [21], and *N, N*‐dimethylformamide (DMF) [20], while sulfur nucleophiles have recently been used to construct C─S bonds to generate hydroxymethanesulfonate (HMS) [14, 15], collectively demonstrating the power of nucleophilic attack as a powerful strategy for steering eCO_2_RR reaction pathways beyond conventional product distributions.

Despite these advances, the direct formation of C─C bonds between CO_2_ reduction intermediates and exogenous carbon nucleophiles remains a fundamentally unresolved challenge. Achieving such reactions would transcend the intrinsic carbon framework dictated by CO_2_ alone and enable the modular construction of expanded carbon skeletons under electrochemical conditions. However, this goal is impeded by the intrinsically low nucleophilicity of carbon‐containing reagents [22], competing reduction reaction [23, 24] and hydrogen evolution reaction (HER) [25, 26], and the transient nature of key CO_2_ reduction intermediates [27].

**SCHEME 1:**
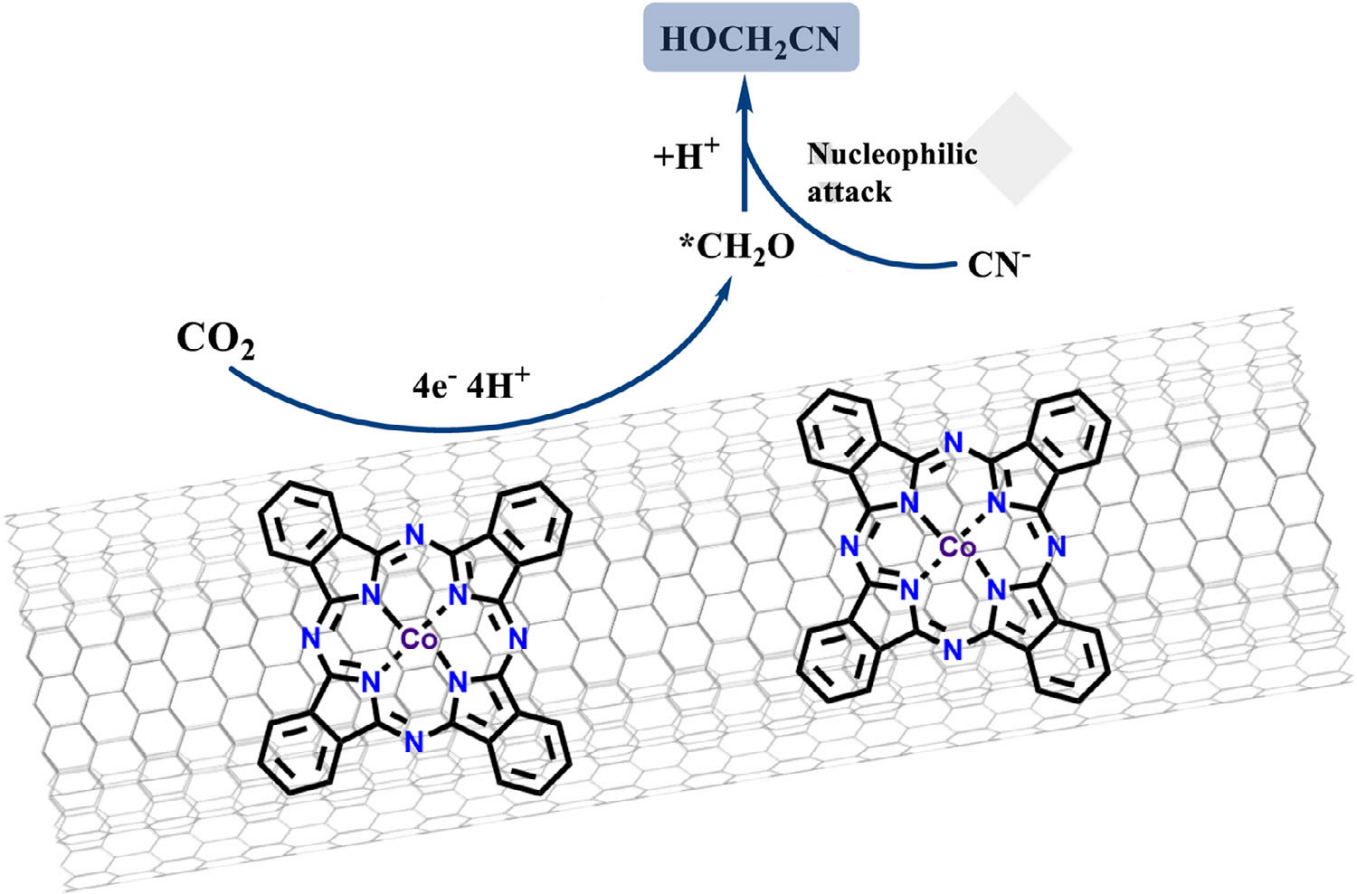
Concept of electrochemical synthesis of glycolonitrile. Demonstration of the C─C bond construction strategy using CO_2_ and cyanide. The product glycolonitrile is a high‐value compound with broad applications and significant industrial potential. Electrolysis was undertaken using CoPc/MWCNT as the catalyst at −1.45 V vs RHE in CO_2_‐saturated 0.1 M K_2_SO_4_ solution containing 60 mM TMSCN at 4°C.

Herein, we address this challenge by using cobalt phthalocyanine supported on multi‐walled carbon nanotubes (CoPc/MWCNT) with well‐defined active sites to elucidate and control the reaction pathway [28, 29]. By integrating experimental investigations with theoretical calculations, we demonstrate for the first time that the exogenous carbon nucleophile cyanide (CN^−^) [30, 31] can efficiently intercept the key C_1_ intermediate *CH_2_O generated during eCO_2_RR, enabling C─C bond formation to produce glycolonitrile with up to 6.6% faradaic efficiency (FE) (Scheme 1).

Cyanide is a highly toxic industrial pollutant commonly generated in metallurgical and electroplating processes [32, 33]. The coupled electrochemical conversion of CO_2_ and cyanide into a high‐value‐added chemical thus offers a unique “waste‐to‐wealth” strategy that integrates carbon utilization with cyanide remediation. Glycolonitrile is an important synthetic precursor for amino acids and diamines, including glycine and ethylenediamine [34, 35]. The glycolonitrile market size was USD 101 million in 2023 and is expected to reach USD 170.64 million by 2032, underscoring the practical relevance of this transformation [36]. Subsequently, hydroxylamine hydrochloride (NH_2_OH•HCl) was introduced into the reaction system, and by integrating a multicomponent reaction with eCO_2_RR, we achieved the electrosynthesis of the biomolecule glycine under ambient pressure. Collectively, this work establishes a previously unexplored reaction manifold for eCO_2_RR by enabling exogenous C─C bond construction, expands the scope of electrochemical CO_2_ upgrading beyond traditional product classes, and provides a conceptual blueprint for using renewable electricity to synthesize structurally complex C‐C frameworks from abundant feedstocks.

## 2 Results and Discussion

This electrochemical C─C bond formation was investigated by controlled potential electrolysis (CPE) in a three‐electrode H‐cell using CoPc/MWCNT as the working electrode. X‐ray absorption near‐edge structure (XANES) and x‐ray absorption fine structure (EXAFS) analyses reveal that no Co─Co bonds are present in CoPc/MWCNT and the peak at 1.46 Å in EXAFS spectrum is attributable to Co‐N coordination, confirming atomic dispersion of Co atoms in CoPc/MWCNT (Figure S1) [37, 38]. The Co(II)/Co(I) redox feature of CoPc/MWCNT was observed in the cyclic voltammogram (Figure S2). CO_2_‐saturated 0.1 M K_2_SO_4_ was used as electrolyte. Gas products were quantified by gas chromatography (GC), and liquid products were identified by ^1^H NMR after electrolysis.

**FIGURE 1:**
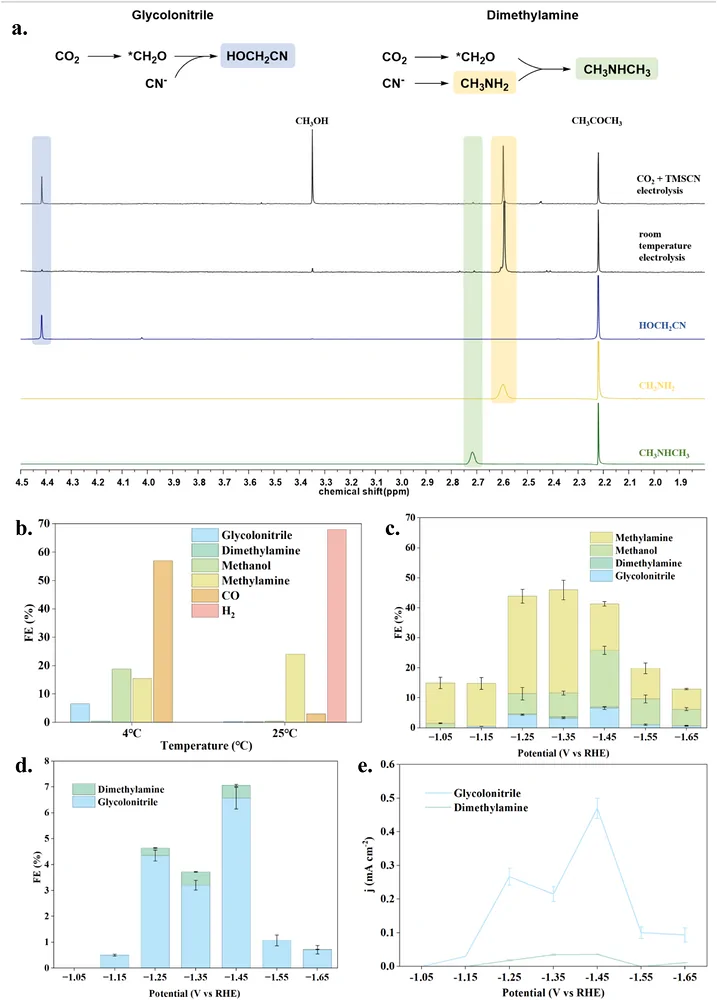
Electrochemical synthesis of glycolonitrile. (a) ^1^H NMR spectra of liquid products obtained from the electrolysis of a CO_2_‐saturated 0.1 M K_2_SO_4_ solution containing 60 mM TMSCN, confirming the formation of methylamine, dimethylamine, and the C‐C coupled product glycolonitrile. The electrolysis was undertaken at ‐1.45 V vs RHE for 7200 s. (b) Product FE was measured after electrolysis at 4°C and at room temperature. The lower temperature effectively suppresses HER, leading to an increased availability of carbon intermediates for coupling. (c‐d) The distribution of FE of the reaction product as a function of applied potential. Cyanide reduction is markedly suppressed at −1.45 V vs RHE due to the interception of carbon‐containing intermediates and enhanced C‐C coupling efficiency. (e) Partial current density of coupling products as a function of applied potential.

Guided by the Linear sweep voltammetry (LSV) results (Figure S3), electrolysis was first undertaken at 4°C and 25°C in a CO_2_‐saturated 0.1 M K_2_SO_4_ solution containing 60 mM trimethylsilyl cyanide (TMSCN) at the potential of −1.45 V vs reversible hydrogen electrode (RHE) (Figure 1). Six products were obtained, including two regular eCO_2_RR products—CO and CH_3_OH—along with H_2_ from the H_2_ evolution reaction (HER), methylamine from the reduction of TMSCN, glycolonitrile, and dimethylamine. The latter is postulated to form via the reaction between methylamine, the product from the reduction of TMSCN as supported by the control experiment undertaken in a N_2_‐saturated 0.1 M K_2_SO_4_ solution containing 60 mM TMSCN (Figures S4 and S5), and a carbon‐containing intermediate from eCO_2_RR (Figure 1a) [16]. The combined FEs for all these products were around 100% (Figure 1b), suggesting that there is no major unidentified product. In eCO_2_RR, H_2_ evolution is inevitable due to the kinetic advantage of this process and abundance of proton sources. Remarkably, lowering the temperature from 25°C to 4°C decreases the FE of the reduction of cyanide and almost completely suppresses H_2_ generation [39, 40]. This temperature effect is consistent with the previous temperature‐dependent CO_2_RR study [41], which showed that lowering the electrolyte temperature can suppress HER and alter CO_2_RR selectivity by regulating intermediate adsorption and the interfacial water microenvironment. Therefore, the use of 4°C provides a more favorable environment for suppressing HER and cyanide reduction, maintaining sufficient cyanide, and promoting their coupling with CO_2_‐derived C_1_ intermediates. In contrast, at room temperature, the FE for HER exceeded 50%, limiting the formation of C_1_ intermediates available for subsequent coupling reactions (Figure 1b). Therefore, in subsequent experiments, we used 4°C unless otherwise specified, as this condition suppressed competing HER and cyanide reduction, allowing the C‐C coupling pathway toward glycolonitrile to be more clearly investigated.

When the applied potential was increased from −1.05 to −1.35 V vs RHE, the reduction of cyanide to methylamine was significantly enhanced, and methylamine became the dominant liquid product. When the potential was further increased to −1.45 V vs RHE, the reduction of cyanide to methylamine drops sharply, which facilitates the progression of eCO_2_RR and the interception of key carbon‐containing intermediates by cyanide. A small amount of the 10e^−^ product dimethylamine was also detected (Figure 1c–e). The relatively high FE of liquid products at −1.25 V and −1.35 V vs RHE mainly arises from cyanide reduction to methylamine rather than from the desired C‐C coupling pathway. Therefore, the potential dependence of glycolonitrile formation does not follow a simple volcano‐shaped trend, but reflects the balance between CO_2_ reduction to reactive C_1_ intermediates, cyanide reduction, HER, and C─C bond formation. These results indicate clear competition between eCO_2_RR and cyanide reduction.

To investigate the C─C coupling reaction between carbon‐containing intermediates and cyanide, electrolysis was also undertaken in a CO_2_‐saturated 0.1 M K_2_SO_4_ solution containing 40 or 80 mM TMSCN. The FE for glycolonitrile obtained in the presence of 40 mM TMSCN is only 2.78%, which is significantly lower than the value of 6.60% obtained when 60 mM TMSCN was present. Further increasing the concentration of TMSCN to 80 mM resulted in an increase in the FE for methylamine, thereby constraining CO_2_ reduction and leading to a concomitant decrease in the FE of glycolonitrile (Figures S6 and S7). We also attempted to adjust the K_2_SO_4_ concentration (Figures S8 and S9), but these measures did not enhance the FE of glycolonitrile. In addition, other electrolytes were examined, including the conventional CO_2_RR electrolyte KHCO_3_, but no improvement in glycolonitrile FE was observed; in particular, KHCO_3_ gave a lower FE than K_2_SO_4_ under otherwise comparable conditions. This lower performance in KHCO_3_ is more likely related to the cyanide distribution in the bicarbonate electrolyte. The more neutral buffered environment may favor a higher fraction of free CN^−^ from TMSCN hydrolysis. Free CN^−^ can compete with CO_2_ activation at CoPc sites and promote cyanide reduction, thereby suppressing the reaction between CO_2_‐derived intermediates and cyanide (Figures S10 and S11).

The metal center in phthalocyanine (Pc) catalysts is known to play a crucial role in eCO_2_RR activity and selectivity [42, 43]. To gain mechanistic insight into the C‐C coupling process, a series of catalysts was examined. When bare MWCNT and metal‐free Pc/MWCNT were used as the catalyst, almost no methanol or methylamine was detected, indicating that the metal center serves as the active site for this reaction. When NiPc/MWCNT was used as the catalyst, only trace glycolonitrile was produced, while CuPc/MWCNT and FePc/MWCNT showed no C‐C coupled products (Figures S12 and S13). This result is consistent with our previous work on CoPc/MWCNT catalyzed CO_2_ to HMS electrosynthesis, where CoPc was proposed to bind CO more strongly than NiPc and CuPc, thereby favoring further reduction beyond CO toward formaldehyde‐related C_1_ intermediates. Therefore, the higher glycolonitrile formation on CoPc/MWCNT is likely associated with its ability to access formaldehyde‐related C_1_ intermediates that can be intercepted by cyanide [44, 45]. In contrast, when *CO forms on the surface of other metal Pc, it tends to be released directly into the solution [46, 47]. Furthermore, since *CO is derived from the further reduction of *COOH, we infer that no C─C bond formation occurs between cyanide and *COOH or *CO [14]. Further control experiments confirmed our inference: stable intermediate HCOONa and CO cannot react directly with cyanide in the presence of CoPc/MWCNT under open‐circuit conditions (Figure S14). These results exclude *COOH and *CO as coupling intermediates and point toward deeper reduction intermediates such as *CHO, *CH_2_O, or formaldehyde in solution. This interpretation is consistent with the previous work by Wang and co‐workers, where CoPc/MWCNT was shown to be one of the rare molecular catalyst systems capable of producing methanol through a formaldehyde‐involved pathway during CO_2_ reduction [48, 49, 50]. Therefore, the higher glycolonitrile formation on CoPc/MWCNT likely arises from its ability to generate formaldehyde‐related C_1_ intermediates that can be intercepted by cyanide.

A series of control experiments was subsequently conducted to further probe the reaction mechanism. In the absence of CO_2_, TMSCN, or CoPc catalyst, no glycolonitrile formation was observed (Figures S4 and S15). Removal of TMSCN from the reaction solution resulted in methanol being the major liquid product, implying that the pathway to glycolonitrile likely shares similarities with methanol formation, possibly diverging from the same intermediate. Subsequently, TMSCN was added to the post‐electrolysis solution from this control experiment and stirred for 1 h. NMR analysis showed that only a small amount of glycolonitrile was generated. This result suggests that coupling involving freely diffusing formaldehyde is unlikely to dominate glycolonitrile formation. Instead, the C‐C coupling more likely occurs in the catalyst surface region, where cyanide can react with short‐lived formaldehyde‐like or adsorbed carbonyl C_1_ intermediates generated during CO_2_ reduction [13]. Lower CoPc/MWCNT loading decreased the glycolonitrile FE, whereas further increasing the loading did not lead to additional improvement (Figures S16 and S17). This result indicates that the glycolonitrile FE does not simply scale with the total catalyst amount. The absence of further improvement at higher loading may be associated with mass‐transport limitations within the thicker catalyst layer [51, 52]. In addition, the glycolonitrile FE was not significantly affected by stirring rate (Figures S18 and S19). These results suggest that glycolonitrile formation is associated with the generation and interception of reactive C_1_ intermediates in the catalyst surface region rather than being dominated by freely diffusing solution intermediates. The operational stability of CoPc/MWCNT was further examined by 6 h electrolysis under the optimized conditions using the H‐cell equipped with an external electrolyte reservoir. The current density remained stable without an obvious decrease throughout the electrolysis. The FE of glycolonitrile remained around 6.5%, corresponding to a glycolonitrile partial current density of approximately 0.43 mA cm^−2^ (Figures S20 and S21). CV integration further showed that 94.6% of the electrochemically active CoPc sites were retained after 6 h electrolysis (Figure S21), supporting the stability of CoPc/MWCNT during the C‐C coupling reaction.

**FIGURE 2:**
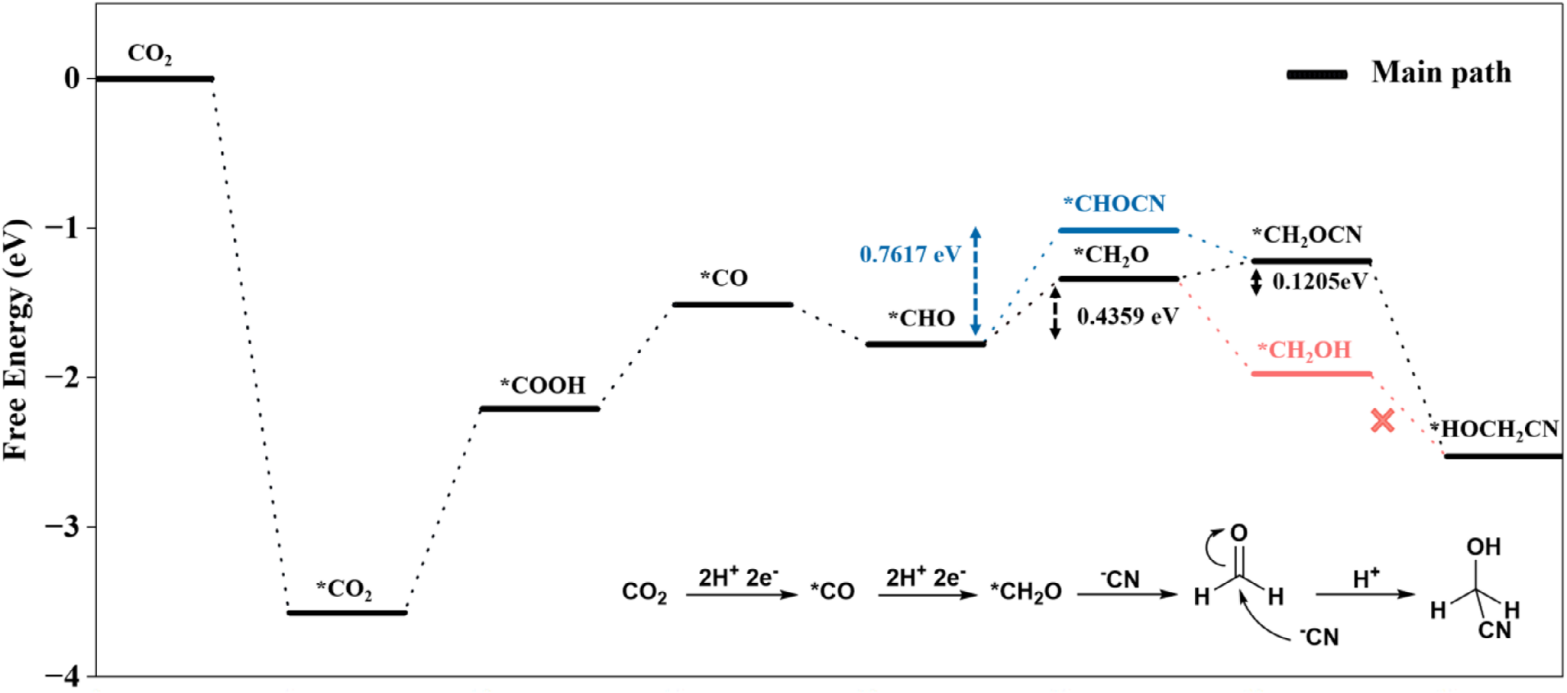
DFT calculations of the free‐energy diagram for the synthesis of glycolonitrile. The black curve represents the primary route for forming the C─C bond. The blue curve represents the formation of *CHOCN. The red curve represents the route for forming methanol.

Density functional theory (DFT) calculations were performed to further elucidate the C‐C coupling mechanism, guided by the experimental observations (Figure 2 and Figure S22). A MWCNT‐supported CoPc model with implicit solvation, as detailed in the Supporting Information, was used to better represent the experimental CoPc/MWCNT catalyst. This modelling strategy is consistent with previous studies on CoPc/CNT‐based catalysts, where the curvature effect of CNTs was considered negligible because the CNT diameter is much larger than the molecular size of CoPc [53].

The calculated free energy for the coupling between cyanide and *CHO is 0.7617 eV, which is higher than that for the further reduction of *CHO to *CH_2_O (0.4359 eV). Accordingly, the most favorable coupling pathway proceeds via *CH_2_O, consistent with intermediates previously proposed in electrocatalytic CO_2_ involved N‐methylation reactions. Although *CH_2_OH is relatively stable in the calculated energy profile, it is not considered the productive C‐C coupling intermediate. After the fifth proton‐electron transfer from *CH_2_O to *CH_2_OH, the carbonyl π acceptor orbital is no longer available. Therefore, *CH_2_OH cannot accept the lone pair from CN^−^ through the same two‐electron nucleophilic addition pathway required for glycolonitrile formation. Although direct spectroscopic detection of *CH_2_O under the present conditions was not achieved, the involvement of formaldehyde‐related intermediates is consistent with previous studies on CoPc/CNT catalyzed CO_2_ to methanol conversion [48, 49, 50]. It is also supported by our previous CoPc/CNT work, in which sulfite trapping was used to detect formaldehyde formation from CO_2_ reduction [44, 45]. Together with the present control experiments and DFT results, these observations support formaldehyde‐related species as the most plausible intermediate intercepted by cyanide. Notably, this coupling mode fundamentally differs from reported C‐C coupling processes in eCO_2_RR that produce C_2+_ products, such as *CO‐*CO or *CO‐*CHO coupling toward ethylene or ethanol [54, 55].

Because the coupling between *CH_2_O and cyanide competes with the further reduction of *CH_2_O to methanol, future efforts should focus on promoting CN^−^ capture at the four‐electron CH_2_O stage while suppressing HER and cyanide reduction. In general, nucleophilic addition to carbonyl compounds is governed by electronic effects, steric hindrance, and nucleophile strength. Therefore, other carbon nucleophiles such as dibenzoylmethane [56] or phenylboronic acid [57] are in principle capable of undergoing similar coupling reactions. However, their poor solubility in aqueous media limits practical implementation. Future work will thus explore non‐aqueous systems to further promote this class of transformations.

Finally, we further expanded the reaction scope [45]. In previous studies on electrocatalytic C─N bond formation, hydroxylamine has been demonstrated as an effective nitrogen source for co‐reduction with CO_2_ to produce methylamine [16]. Therefore, we hypothesized that the imine intermediate formed during the reduction of hydroxylamine and CO_2_ could be intercepted by cyanide in our system, enabling a *Strecker* reaction (Figure S23) to generate aminoacetonitrile, which could subsequently hydrolyze to glycine.

**FIGURE 3:**
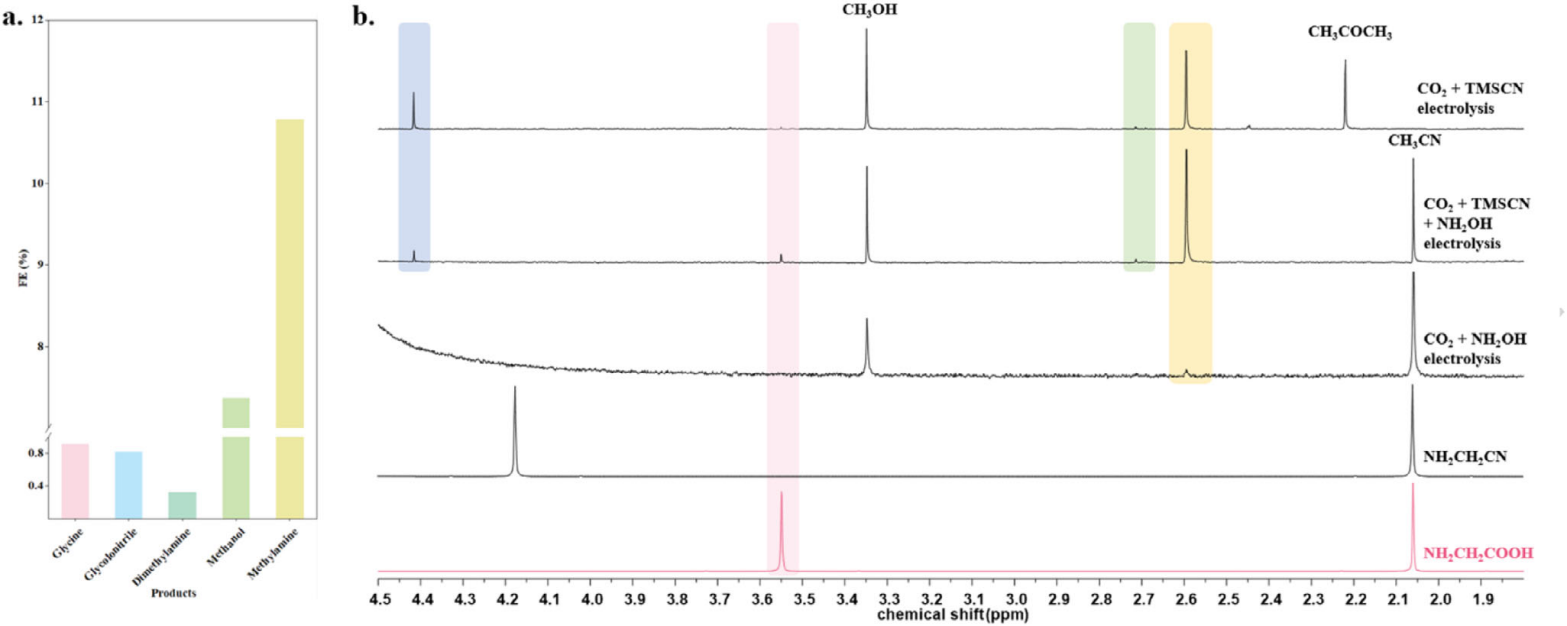
Preparation of glycine by a three‐component electrocatalytic reaction. (a) FE distribution of liquid products obtained from the electrocatalytic three‐component reaction of TMSCN, NH_2_OH, and CO_2_ for glycine synthesis. (b) ^1^H NMR spectra of the TMSCN‐NH_2_OH‐CO_2_ system after electrolysis, control experiments, and a glycine reference.

Accordingly, 50 mM NH_2_OH•HCl was introduced into the catholyte, while all other reaction conditions were kept identical (i.e. −1.45 V vs RHE, 60 mM TMSCN, 0.1 M K_2_SO_4_, 7200 s). Prior to electrolysis, a few drops of 1 M KOH solution were added to adjust and maintain the initial pH. ^1^H NMR analysis revealed the formation of glycine with a FE of 0.9% (Figure 3). The result of the control experiment (Figure S24) shows that when glycolonitrile and NH_2_OH were used as reactants in electrolysis, no formation of glycine was observed, indicating that glycine is not formed by the direct attack of NH_2_OH on glycolonitrile followed by hydrolysis. Notably, this electrocatalytic multicomponent reaction circumvents the high‐pressure conditions required in previous electrocatalytic CO_2_ to amino acid synthesis [19]. In calculating the FE in this section, glycine formation was treated as a six‐electron process, consisting of a four‐electron reduction of CO_2_ to formaldehyde followed by a two‐electron N─O bond cleavage step, consistent with previous CoPc/CNT‐catalyzed CO_2_/NH_2_OH coupling studies [16]. Methylamine was assigned to originate predominantly from the four‐electron reduction of cyanide rather than from an eight‐electron co‐reduction of NH_2_OH and CO_2_, because control experiments revealed that the co‐reduction of NH_2_OH and CO_2_ was inefficient under our reaction conditions. Consequently, the insufficient formation of the key imine intermediate (Figure 3b) derived from NH_2_OH‐CO_2_ co‐reduction constitutes the primary reason for the currently low FE of glycine. We subsequently adjusted the reactant concentrations by increasing the NH_2_OH•HCl concentration to 100 mM and decreasing the TMSCN concentration to 30 mM. Under these conditions, only trace amounts of glycolonitrile were detected, while the FE of glycine increased to 2.8% (Figure S25). More broadly, this reaction scheme provides an alternative strategy for introducing complex functional groups into eCO_2_RR, enabling the electrosynthesis of high value‐added molecules [45].

## 3 Conclusion

In summary, we have demonstrated a previously unexplored electrocatalytic route for constructing C─C bonds during CO_2_ reduction through the interception of deeply reduced C_1_ intermediates by an exogenous carbon nucleophile. This strategy bypasses conventional coupling pathways and enables the selective synthesis of glycolonitrile on CoPc/MWCNT catalyst. Mechanistic studies reveal *CH_2_O as the main coupling intermediate, while temperature regulation suppresses competing reactions to enhance efficiency. Extending this concept to a multicomponent system further enables direct electrosynthesis of glycine from CO_2_ under ambient conditions. Beyond providing a new reaction route for eCO_2_RR, this work offers a general strategy for constructing structurally complex carbon frameworks from simple and abundant feedstocks using renewable electricity.

## Author Contributions

**Gongbo Liu**: conceptualization, methodology, formal analysis, investigation, validation, data curation, Writing – original draft, Writing – review and editing. **Liuru Fang**: software, data curation, writing – original draft, writing – review and editing. **Dayu Zhu**: writing – review and editing, writing – original draft, validation, methodology, data curation. **Aimin Li**: software, writing – review and editing, writing – original draft. **Haoming Yu**: writing – review and editing, writing – original draft, methodology. **Chunlu Ding**: writing – original draft, writing – review and editing, methodology. **Yuen‐Leong Chow**: methodology, writing – review and editing, writing – original draft. **Mengjie Liu**: methodology, writing – review and editing, writing – original draft. **Jianqi Geng**: methodology, writing – review and editing, writing – original draft. **San H. Thang**: supervision, writing – review and editing, writing – original draft. **Jie Zhang**: supervision, writing – original draft, writing – review and editing, funding acquisition, resources, project administration, conceptualization.

## Conflicts of Interest

The authors declare no conflicts of interest.

## Supporting information




**Supporting File**: advs77346‐sup‐0001‐SuppMat.docx.

## Data Availability

The data that support the findings of this study are available from the corresponding author upon reasonable request.
