# Supplementary material for: Electrocatalytic C─C Bond Formation Via Cyanide Interception of CO2 Reduction Intermediates
Source: Adv Sci (Weinh). 2026 Aug 21:e77346. Online ahead of print. doi: 10.1002/advs.77346 (PMC13496289; doi:10.1002/advs.77346)
Supplement: Supplementary file 1 [file ADVS-9999-e77346-s001.docx]

**Electrocatalytic C-C Bond Formation via Cyanide Interception of CO_2_ Reduction Intermediates**

Gongbo Liu^a^, Liuru Fang^a^, Dayu Zhu^a^, Aimin Li^a^, Haoming Yu^a^, Chunlu Ding^a^, Yuen-Leong Chow^a^, Mengjie Liu^a^, Jianqi Geng^b^, San H. Thang^a^, and Jie Zhang^a, c, d,*^

^a^ School of Chemistry, Monash University, Clayton, Melbourne, Victoria 3800, Australia

^b^ School of Chemistry and Chemical Engineering, Beijing Institute of Technology, Beijing, 102488, China

^c^ ARC Research Hub for Carbon Utilisation and Recycling, Monash University, Clayton, VIC 3800, Australia

^d^ ARC Centre of Excellence for Green Electrochemical Transformation of Carbon Dioxide, Monash University, Clayton, VIC 3800, Australia

email address: jie.zhang@monash.edu

# **Table of contents:**

[1. Materials:	1](#_Toc233061820)

[2. Catalyst Preparation:	2](#_Toc233061821)

[3. Controlled potential electrolysis:	3](#_Toc233061822)

[4. Product quantification:	4](#_Toc233061823)

[5. DFT calculation:	5](#_Toc233061824)

[6. Supplementary Figures:	6](#_Toc233061825)

[Figure S1 EXAFS spectra in R space (a) and XANES spectra (b).	6](#_Toc233061826)

[Figure S2 Cyclic voltammogram of CoPc/MWCNT.	7](#_Toc233061827)

[Figure S3 Linear sweep voltammetry.	8](#_Toc233061828)

[Figure S4 ^1^H NMR spectra of the control experiments.	9](#_Toc233061829)

[Figure S5 ^1^H NMR calibration curves.	10](#_Toc233061830)

[Figure S6 Different concentrations of TMSCN in the electrocatalytic reaction.	11](#_Toc233061831)

[Figure S7 ^1^H NMR results of experiments with different TMSCN concentrations.	12](#_Toc233061832)

[Figure S8 Different concentrations of K_2_SO_4_ in the electrocatalytic reaction.	13](#_Toc233061833)

[Figure S9 ^1^H NMR results of experiments with different concentrations of K_2_SO_4_.	14](#_Toc233061834)

[Figure S10 Different electrolytes in the electrocatalytic reaction.	15](#_Toc233061835)

[Figure S11 ^1^H NMR results of experiments with different electrolytes.	16](#_Toc233061836)

[Figure S12 The performance of different catalysts in the electrocatalytic reaction.	17](#_Toc233061837)

[Figure S13 ^1^H NMR results of experiments with different catalysts.	18](#_Toc233061838)

[Figure S14 ^1^H NMR results of some control experiments.	19](#_Toc233061839)

[Figure S15 The FE of glycolonitrile in control experiments.	20](#_Toc233061840)

[Figure S16 Different catalyst loading in the electrocatalytic reaction.	21](#_Toc233061841)

[Figure S17 ^1^H NMR results of experiments with different catalyst loading.	22](#_Toc233061842)

[Figure S18 Different stirring rate in the electrocatalytic reaction.	23](#_Toc233061843)

[Figure S19 ^1^H NMR results of experiments with different stirring rate.	24](#_Toc233061844)

[Figure S20 Photograph of the long term electrolysis.	25](#_Toc233061845)

[Figure S21 CVs of the CoPc/MWCNT catalyst before and after the reaction.	26](#_Toc233061846)

[Figure S22 Modelling of possible intermediates.	27](#_Toc233061847)

[Figure S23 The classic *Strecker* amino acid (e.g., glycine) synthesis mechanism.	28](#_Toc233061848)

[Figure S24 ^1^H NMR results of control experiments to generate glycine.	29](#_Toc233061849)

[Figure S25 ^1^H NMR results of optimized glycine synthesis.	30](#_Toc233061850)

[7. References:	31](#_Toc233061851)

# 1. Materials:

All chemicals were purchased and used as received unless otherwise stated. Cobalt(II) phthalocyanine (CoPc, ≥97.0 %), Iron(II) phthalocyanine (FePc, ≥97.0 %), Copper(II) phthalocyanine (CuPc, ≥97.0 %), Nickel(II) phthalocyanine (NiPc, ≥97.0 %), phthalocyanine (Pc, ≥97.0 %), ethanol (99.7%), acetone (≥99.0%), hydroxylamine hydrochloride (99.0%), and dimethylformamide (DMF, 99.9%) were supplied by Merck. Deuterium oxide (99.9 atom % D), Glycolonitrile (70% in water), formaldehyde solution (contains 10-15% methanol as stabilizer, 37 wt. % in H_2_O) and Nafion (5 wt %) were purchased from Sigma-Aldrich. Potassium sulfate (K_2_SO_4_, 99.9%) was procured from UNIVAR. Multi-walled carbon nanotubes GT-300 (MWCNT) were purchased from Dazhan Nanometer. Carbon paper (AvCarb MB30) was purchased from Fuel Cell Store. Trimethylsilyl cyanide (TMSCN, 96%) was purchased from Energy. High-purity nitrogen gas (99.999%) and carbon dioxide (>99%, Aligal) were provided by Air Liquide, Australia. Ultrapure water (18.25 MΩ cm at 25℃) from Milli-Q^®^ Direct Water Purification System (Merck) was used in all experimental processes.

# 2. Catalyst Preparation:

20 mg MWCNT (MWCNT, MWCNT-COOH, MWCNT-NH_2_) were subsequently dispersed in 10 ml DMF using sonication for 1h. Then, 4 mg MPc (CoPc, FePc, NiPc, CuPc) or Pc were added to the MWCNT suspension and sonicated for 1h to obtain a well-mixed suspension, which was further stirred at room temperature for 24 h. Subsequently, the mixture was centrifuged and the precipitate was washed with DMF and ethanol. Finally, the precipitate was lyophilized to yield the final product.

Catalyst ink was prepared by dispersing 2 mg of hybrid materials (MPc/MWCNT, Pc/MWCNT, CoPc /MWCNT-COOH, or CoPc/MWCNT-NH_2_) in 2 mL of ethanol with 12 µL of 5 wt% Nafion solution, followed by sonication for 1 h. 200 µL of the ink was then drop-casted onto a 2.5 × 1 cm^2^ polytetrafluoroethylene-treated carbon fibre paper to cover an area of 1 × 1 cm^2^ (catalyst mass loading, 0.2 mg cm^-2^). The prepared electrodes were fully dried using an infrared lamp.

# 3. Controlled potential electrolysis:

All electrochemical measurements were conducted in a three-electrode system using an electrochemical workstation (CHI760D, CH instruments) and a custom-made gas-tight two-compartment H-cell. Each compartment contained 10 mL of electrolyte and 40 mL of gas headspace. Carbon rod and Ag/AgCl (1 M KCl) were used as the counter electrode and reference electrode, respectively. All potentials measured in this work were converted to RHE by the following equation:

E (vs RHE) = E (vs Ag/AgCl) + 0.222 + (0.059 × pH)

0.1 M K_2_SO_4_ with different concentrations of TMSCN was used as the electrolyte. Linear sweep voltammetry (LSV) was measured in the range of 0.55 ~ -1.85 V (vs. RHE), with a sweep rate of 0.05 V s^-l^. Cyclic voltammograms (CV) was measured in the range of 0.7 ~ -0.5 V (vs. RHE), with a sweep rate of 0.05 V s^-l^. Potentiostatic electrolysis was performed at room pressure and 4 ℃, with a steady CO_2_ gas flow which is 10 mL min^-1^.

# 4. Product quantification:

For gas product analysis, all gas products were examined using a gas chromatograph (GC) equipped with a thermal conductivity detector (TCD). TCD was used to identify H_2_ and CO, which were separated using an HP-Plot Q column. The carrier gas is N_2_.

Liquid products of electrocatalysis were detected using a Bruker 400 MHz NMR spectrometer. NMR samples were prepared by adding 400 μL of electrolyte taken from the cathode compartment immediately post-electrolysis into an NMR tube containing 50 μL of an internal standard (5 mM acetone or 5 mM acetonitrile) dissolved in ultrapure water and then adding 100 μL D_2_O. Faradaic efficiency (FE) was calculated by the following formula:

$$FE=\frac{N\times F\times C\times V}{Q}$$

Where N represents the number of electrons transferred; F is the Faraday constant (96485.3 C mol^−1^); C is the measured product molar concentrations; V is the total volume of the electrolyte, Q is the total charge passed through the working electrode.

# 5. DFT calculation:

The slab models were constructed in an 17.22 × 17.04 × 30.00 Å^3^ simulation cell with two-dimensional periodic boundary conditions (x, y) and Γ-point sampling. The exchange correlation functional of Perdew, Burke, and Ernzerhof (PBE) was used [1]. DFT calculations are all performed using the CP2K code [2] employing the Gaussian and plane-wave (GPW) scheme and the QUICKSTEP module. Core electrons were described by GTH-PBE pseudopotentials in combination with DZVP-MOLOPT-SR-GTH basis sets for all elements [3]. Dispersion interactions were accounted for by Grimme’s D3(BJ) correction. Implicit solvation was included to account for bulk solvent screening effects using the self‑consistent continuum solvation model with the Andreussi method and a dielectric constant of 78.40[4]. The plane-wave auxiliary grid was set by a density cutoff of 550 Ry and a relative cutoff of 60 Ry. Electrostatics for the slab geometry were handled using the Martyna–Tuckerman Poisson solver. Self-consistent-field (SCF) calculations were converged to 1.0 × 10^−6^.

To compute energy differences of elementary proton coupled electron transfer (PCET) steps, the computational hydrogen electrode (CHE) model [5] was used. In this model we assume hydrogen gas is at equilibrium with proton and electron and the corresponding potential is 0 V vs. RHE, thus the energy of proton coupled with electron is estimated by half of the energy of hydrogen gas:

H^+^ + e^−^ ↔ H_2_ (g) / 2

E_(H^+^ + e^−^) = E_(H_2_ (g))/2, @ pH = 0 and 1 atm.

# 6. Supplementary Figures:

### Figure S1 EXAFS spectra in R space (a) and XANES spectra (b).

### Figure S2 Cyclic voltammogram of CoPc/MWCNT.

The redox couple centered at ∼0.1 V vs RHE is attributed to the Co (II)/Co (I) feature of the CoPc molecule [6, 7].

### Figure S3 Linear sweep voltammetry.

From the LSV, the presence of TMSCN slightly increases the overall current density in the negative potential region. Scan rate = 0.05 V s^-l^.

### Figure S4 ^1^H NMR spectra of the control experiments.

^1^H NMR spectra of liquid products obtained from the control experiments. All reactions were performed at 4 ℃.

Before the reaction: 60 mM TMSCN was added into CO_2_ saturated 0.1 M K_2_SO_4_ solution without electrolysis.

CN^-^ to CH_3_NH_2_: 60 mM TMSCN was added into N_2_-saturated 0.1 M K_2_SO_4_ solution. The electrolysis was undertaken at -1.45 V vs RHE for 7200 s.

CO_2_ to CH_3_OH: the electrolysis was undertaken at -1.45 V vs RHE for 7200 s in a CO_2_-saturated 0.1 M K_2_SO_4_ solution.

After CO_2_ to CH_3_OH added TMSCN: 60 mM TMSCN was added after CO_2_ to CH_3_OH reaction.

CH_3_OH + TMSCN electrolysis: 50 mM CH_3_OH and 60 mM TMSCN electrolysis was added into N_2_-saturated 0.1 M K_2_SO_4_ solution in anode side. The electrolysis was undertaken at -1.45 V vs RHE for 7200 s.

### Figure S5 ^1^H NMR calibration curves.

(a) glycolonitrile; (b) dimethylamine; (c) methanol; (d) methylamine.

### Figure S6 Different concentrations of TMSCN in the electrocatalytic reaction.

Different concentration of TMSCN was added into CO_2_-saturated 0.1 M K_2_SO_4_ solution. The electrolysis was undertaken at -1.45 V vs RHE and 4 °C for 7200 s.

### Figure S7 ^1^H NMR results of experiments with different TMSCN concentrations.

^1^H NMR spectra of liquid products obtained from the experiments with different TMSCN concentrations.

### Figure S8 Different concentrations of K_2_SO_4_ in the electrocatalytic reaction.

60 mM TMSCN was added into CO_2_-saturated K_2_SO_4_ solution. The electrolysis was undertaken at -1.45 V vs RHE and 4 °C for 7200 s.

### Figure S9 ^1^H NMR results of experiments with different concentrations of K_2_SO_4_.

^1^H NMR spectra of liquid products obtained from the experiments with different concentrations of K_2_SO_4_.

### Figure S10 Effect of electrolytes in the electrocatalytic reaction.

60 mM TMSCN was added into CO_2_-saturated 0.1 M electrolyte solution. The electrolysis was undertaken at -1.45 V vs RHE and 4 °C for 7200 s.

### Figure S11 ^1^H NMR results of experiments with different electrolytes.

^1^H NMR spectra of liquid products obtained from the experiments with different electrolytes.

### Figure S12 The performance of different catalysts in the electrocatalytic reaction.

60 mM TMSCN was added into CO_2_-saturated 0.1 M K_2_SO_4_ solution. The electrolysis was undertaken at -1.45 V vs RHE and 4 °C for 7200 s.

### Figure S13 ^1^H NMR results of experiments with different catalysts.

^1^H NMR spectra of liquid products obtained from the experiments with different catalysts.

### Figure S14 ^1^H NMR results of some control experiments.

^1^H NMR spectra of liquid products obtained from the control experiments to generate glycolonitrile. 0.1 M HCOONa, 0.1 M HCHO, and a saturated CO solution were used as reactants and reacted with TMSCN, respectively. The solution also contained 0.1 M K_2_SO_4_; an appropriate amount of concentrated H_2_SO_4_ was added to adjust the pH to 4. CoPc/MWCNT was used as the catalyst under open-circuit conditions. The reaction was conducted at 4°C for 7200 s. HCOONa and CO can not react with TMSCN, whereas HCHO can react with TMSCN to form glycolonitrile.

### Figure S15 The FE of glycolonitrile in control experiments.

The reaction conditions without CO_2_ and without TMSCN are the same as CN^-^ to CH_3_NH_2_ and CO_2_ to CH_3_OH in Figure S4, respectively. Without catalyst: 60 mM TMSCN was added into CO_2_-saturated 0.1 M K_2_SO_4_ solution. The electrolysis was using carbon paper as a catalyst and undertaken at -1.45 V vs RHE and 4 °C for 7200 s.

### Figure S16 Different catalyst loading in the electrocatalytic reaction.

60 mM TMSCN was added into CO_2_ saturated 0.1 M K_2_SO_4_ solution. The electrolysis was undertaken at -1.45 V vs RHE and 4 °C for 7200 s. The catalyst loadings were 0.15 mg*cm^-2^, 0.2 mg*cm^-2^, and 0.25 mg*cm^-2^, respectively.

### Figure S17 ^1^H NMR results of experiments with different catalyst loading.

^1^H NMR spectra of liquid products obtained from the experiments with different catalyst loading.

### Figure S18 Different stirring rate in the electrocatalytic reaction.

60 mM TMSCN was added into CO_2_-saturated 0.1 M K_2_SO_4_ solution. The electrolysis was undertaken at -1.45 V vs RHE and 4 °C for 7200 s. The standard stirring rate is about 400 revolutions per minute (rpm), the low stirring rate is about 200 rpm, and the high stirring rate is about 600 rpm.

### Figure S19 ^1^H NMR results of experiments with different stirring rate.

^1^H NMR spectra of liquid products obtained from the experiments with different stirring rate.

### Figure S20 Photograph of the long-term electrolysis.

60 mM TMSCN was added to a CO_2_-saturated 0.1 M K_2_SO_4_ solution. The electrolysis was undertaken at -1.45 V vs RHE and 4 °C. (a) Photograph of the experimental configuration used for long-term electrolysis. (b) The i-t data for 6h electrocatalytic reaction. (c) The FE of glycolonitrile for 6h electrocatalytic reaction.

### Figure S21 CVs of the CoPc/MWCNT catalyst before and after the reaction.

The CoPc loading and the amount of electrochemically accessible CoPc before and after electrolysis were evaluated by ICP-MS analysis and CV integration. ICP-MS showed that the Co content in CoPc/MWCNT was approximately 1.6 wt%. Based on the theoretical Co mass fraction in CoPc of 10.3 wt%, the CoPc content in the composite was estimated to be approximately 15.0 wt%. With a catalyst loading of 0.20 mg cm⁻², the total CoPc loading on the electrode was calculated to be approximately 30.0 μg cm⁻². The electrochemically accessible CoPc loading was then estimated by integrating the Co(II)/Co(I) redox feature in the CV curves recorded before and after electrolysis. The integrated charge decreased only slightly from 3.75 × 10⁻³ C before electrolysis to 3.56 × 10⁻³ C after 6 h electrolysis, corresponding to electrochemically accessible CoPc loadings of 22.3 and 21.1 μg cm⁻², respectively. These values account for 74.2% and 70.2% of the total CoPc loading before and after electrolysis, corresponding to 94.6% retention of electrochemically accessible CoPc sites. These results indicate that most CoPc sites remained electrochemically accessible after electrolysis, supporting the stability of CoPc/MWCNT under the reaction conditions.

### Figure S22 Modelling of possible intermediates.

The possible adsorption configuration diagrams of reactants, intermediates, and products involved in the C-C coupling on the CoPc/MWCNT.

### Figure S23 The classic *Strecker* amino acid (e.g., glycine) synthesis mechanism.

### Figure S24 ^1^H NMR results of control experiments to generate glycine.

100 mM glycolonitrile and 100 mM NH_2_OH were added into N_2_-saturated 0.1 M K_2_SO_4_ solution. The electrolysis was undertaken at -1.45 V vs RHE and 4 °C for 7200 s. No glycine was detected, so under our reaction conditions, glycine is not formed by direct attack of NH_2_OH on glycolonitrile followed by hydrolysis.

### Figure S25 ^1^H NMR results of optimized glycine synthesis.

30 mM TMSCN and 100 mM NH_2_OH were added into CO_2_-saturated 0.1 M K_2_SO_4_ solution. The electrolysis was undertaken at -1.45 V vs RHE and 4 °C for 7200 s.

# 7. References:

[1] J. P. Perdew, K. Burke, M. Ernzerhof, *Phys. Rev. Lett.* **1996**, *77*, 3865-3868.

[2] J. VandeVondele, M. Krack, F. Mohamed, M. Parrinello, T. Chassaing, J. Hutter, *Comput. Phys. Commun.* **2005**, *167*, 103-128.

[3] J. VandeVondele, J. Hutter, *J. Chem. Phys.* **2007**, *127*, 114105.

[4] O. Andreussi, I. Dabo, N. Marzari, *J. Chem. Phys.* **2012**, *136,* 064102.

[5] J. K. Nørskov, J. Rossmeisl, A. Logadottir, L. Lindqvist, J. R. Kitchin, T. Bligaard, H. Jónsson, *J. Phys. Chem. B* **2004**, *108*, 17886-17892.

[6] B.-H. Lee, H. Shin, A. S. Rasouli, H. Choubisa, P. Ou, R. Dorakhan, I. Grigioni, G. Lee, E. Shirzadi, R. K. Miao, J. Wicks, S. Park, H. S. Lee, J. Zhang, Y. Chen, Z. Chen, D. Sinton, T. Hyeon, Y.-E. Sung, E. H. Sargent, *Nat. Catal.* **2023**, *6*, 234-243.

[7] Q. Zhu, C. L. Rooney, H. Shema, C. Zeng, J. A. Panetier, E. Gross, H. Wang, L. R. Baker, *Nat. Catal.* **2024**, *7*, 987-999.
